# Identification of novel genetic variants associated with feline cardiomyopathy using targeted next-generation sequencing

**DOI:** 10.1038/s41598-025-87852-5

**Published:** 2025-01-31

**Authors:** Jade Raffle, Jose Novo Matos, Marsha Wallace, Lois Wilkie, Richard J. Piercy, Perry Elliott, David J. Connolly, Virginia Luis Fuentes, Androniki Psifidi

**Affiliations:** 1 Clinical Science and Services, Royal Veterinary College, London, UK; 2 Institute of Cardiovascular Science, University College London, London, UK

**Keywords:** Cardiology, Genetics, Genomics

## Abstract

Cardiomyopathies are the most common heritable heart diseases in cats and humans. This study aimed to identify novel genetic variants in cats with hypertrophic cardiomyopathy (HCM) and restrictive cardiomyopathy (RCM) using a targeted panel of genes associated with human cardiomyopathy. Cats were phenotyped for HCM/RCM by echocardiography ± necropsy. DNA was extracted from residual blood, and targeted next-generation sequencing was performed on two separate feline cohorts: an across-breed cohort (23 healthy cats and 21 HCM-affected pedigree or Domestic Shorthair cats), and a within-breed cohort of Birman pedigree cats (14 healthy, 8 HCM-affected, and 6 RCM-affected). Genome Analysis Toolkit was used for variant discovery. Genomic association analyses, including the covariates breed, age, and sex, were conducted to identify genetic variants of interest. We identified genetic variants associated with both HCM and RCM susceptibility in the sarcomeric genes *ACTC1*, *ACTN2*, *MYH7*, *TNNT2* and the non-sarcomeric gene *CSRP3* in the Birman pedigree cats. These findings suggest that, as proposed in humans, there is at least partial overlap in the genetic background between the HCM and RCM phenotypes in cats. These findings offer potential insights for comparative cardiac research and translational medicine.

**Supplementary Information:**

The online version contains supplementary material available at 10.1038/s41598-025-87852-5.

## Introduction

Cardiomyopathies are diseases of the myocardium that can lead to a range of functional and structural abnormalities of the heart. Cardiomyopathies in both humans and cats are associated with elevated risks of congestive heart failure, cardioembolic events, and sudden cardiac death^1–3^. Subcategorization of cardiomyopathies is based on the underlying disease cause, cardiac morphology and function, clinical presentation, and associated signs.

Hypertrophic cardiomyopathy (HCM) is defined in both humans and cats as the presence of a hypertrophied left ventricle (LV) in the absence of abnormal loading conditions capable of producing a similar degree of hypertrophy, such as other cardiac or systemic diseases^3,4^. Human HCM is the most common genetic cardiovascular disease, affecting 0.2% of the global population (1 in 500 individuals)^5^. The inheritance pattern in 60% of cases is autosomal dominant^6^. Nevertheless, more recent findings propose that human HCM follows a more complex mode of inheritance^7^, with reports of genotype-positive/phenotype-negative individuals and a higher estimated population prevalence of 0.5% (1 in 200 individuals)^8^. Among domesticated animals, cats are particularly predisposed to HCM, with an estimated prevalence of 15% reported in the general cat population^9^.

In humans, HCM has a well-characterised genetic basis with more than 1500 variants described to date^10^. In up to 60% of human cases, HCM is caused by variants in genes encoding cardiac sarcomeric proteins that form the cardiac contractile unit; thus, HCM is generally considered a ‘disease of the sarcomere’^11^. Variants in the genes encoding beta-myosin heavy chain (*MYH7*) and myosin-binding protein C (*MYBPC3*) account for the majority of cases in humans (approximately 70% of patients with sarcomeric variants)^11,12^. Less commonly affected genes include cardiac troponin I (*TNNI3*) and cardiac troponin T (*TNNT2*), α-tropomyosin *(TPM1*), myosin light chain (regulatory *MYL2* and essential *MYL3*) and cardiac actin (*ACTC*)^11–13^.

To date, four variants associated with feline HCM susceptibility have been reported within specific cat breeds. Three of these variants are exonic and located in sarcomeric genes: *MYBPC3* in Maine Coon^14^ and Ragdoll cats^15^, and *MYH7* in a non-pedigree domestic shorthair cat (DSH)^16^. The fourth variant, reported in Sphynx cats, is in the non-sarcomeric Alstrom syndrome 1 *(ALMS1)* gene^17^, which is associated with cardiac development and cell regulation^18^. One further intronic (splicing) variant within the sarcomeric *TNNT2* gene was initially associated with HCM in the Maine Coon breed^19^. However, a more recent study found that this variant was present in high frequencies across multiple cat breeds, suggesting that it might not have a significant association with HCM^20^. A recent classification study applying modified ACMG guidelines classified the two *MYBPC3* gene variants A31P and R820W as pathogenic; the *MYH7* E188K variant as likely pathogenic, and the remaining variants of unknown significance^21^. Based on these findings, routine genetic testing is currently recommended only for specific variants in Maine Coon and Ragdoll breeds.

In humans, HCM is most commonly associated with exonic variants in sarcomeric genes^22^. These variants cause variable degrees of left ventricular hypertrophy (LVH) and different cardiomyopathy phenotypes within the same family^23,24^. Individuals sharing the same causative variant may variously exhibit HCM, RCM or dilated cardiomyopathy (DCM) phenotypes^23,24^. This pleiotropy leading to varying disease severity/expression in cardiomyopathies suggests that factors beyond the sarcomeric variant itself may influence disease expression, such as modifier genes, environmental factors, and epigenetic modifiers^13,25^. The non-coding genome is an emerging area of study for HCM, with regulatory variants now identified in human HCM^26,27^. For most variants, the exact underlying molecular mechanisms linking genotype to phenotype remain unclear.

There are numerous similarities between human and feline HCM, highlighting the potential value of using cats as a model to study the human disease. In both species, the disease is spontaneous, exhibiting similar natural histories and wide phenotypic spectra. They also share genetic homology: Orthologous variants associated with HCM in humans have been identified in cats. Specifically, the R820W variant in the *MYBPC3* gene in Ragdolls and the E188K variant in the *MYH7* gene in a DSH, correspond to known human HCM variants^16,28^. Studies of feline HCM could uncover further shared genetic variants, disease mechanisms and therapeutic targets. A better understanding of the genetic factors causing disease and influencing disease severity in cats could aid with diagnosis and management of the disease.

Our main aim was to identify novel genetic variants for HCM susceptibility within and across cat breeds. Our secondary aim was to investigate different forms of cardiomyopathies (HCM and RCM) within a cohort of related and unrelated Birman cats to identify family-specific variants and explore possible co-occurrence of cardiomyopathies within feline families.

HCM has been reported to affect both pedigree and non-pedigree cats, with some breeds displaying a greater predisposition to the disease than others^29^. We initially investigated the disease across multiple pedigree and non-pedigree cats to capture associations across a spectrum of cat breeds. In the second study we focussed on cats of the Birman breed, since Birmans are recognised as a breed with a familial tendency to develop HCM (unpublished observations). We primarily focussed on investigating HCM, since this is the most common cardiomyopathy observed in cats, however we also included Birman cats with RCM. We aimed to determine whether shared HCM/RCM phenotype causative variants exist in Birman cats as they do in humans^23,24^.

## Results

### Descriptive statistics of the studied cat populations

Descriptive statistics of the clinical and echocardiographic characteristics of the Across-breeds cat cohort (*n* = 44) and Birman cat cohort (*n* = 28) are summarised in Tables 1 and 2, respectively. Due to sample limitations, we included two DSH control cats with a left ventricular wall thickness (LVWT) = 5.5 mm. Both cats were from a geriatric population (≥ 9 years old) with no previous signs of heart disease, all other cats included in the control population had an LVWT < 5.5 mm.

**Table 1:** Descriptive statistics of clinical and echocardiographic characteristics of the across-breeds cat cohort aiming to identify genetic variants for HCM susceptibility using a targeted cardiomyopathy gene panel.

	Controls (*n* = 23)	HCM (*n* = 21)
Age (years)	12 [9.0–17.0]	6.9 [1.8–20.0]
Weight (kg)	4.2 [2.41–5.65]	4.8 [3.00–8.55]
Sex: males (%)	8 (35%)	14 (67%)
*Echocardiography*		
LVWT (mm)	4.8 [4.0–5.5]	7.6 [5.7–11.6]
LAD (mm)	14.1 [11.5–17.0]	19.15 [11.2–31.0]
LA/Ao	1.25 [1.0–1.5]	1.8 [1.1–2.7]

Results are presented as median [range] for each variable for the hypertrophic cardiomyopathy (HCM) and control cats. Abbreviations: LAD, left atrial diameter; LA/Ao, left atrium to aorta ratio; LVWT, left ventricular wall thickness at end-diastole.

**Table 2:** Descriptive statistics of clinical and echocardiographic characteristics of the Birman pedigree cat cohort aiming to identify genetic variants for HCM susceptibility using a targeted cardiomyopathy gene panel.

	Controls (*n* = 14)	HCM (*n* = 8)	RCM (*n* = 6)
Age (years)	11.3 [9.2–17.0]	8.35 [1.4–16.4]	8.25 [4.0–17.9]
Weight (kg)	3.53 [2.63–5.20]	4.15 [3.50–5.20]	4.4 [3.50–5.20]
Sex: males (%)	3 (21%)	6 (75%)	3 (50%)
*Echocardiography*			
LVWT (mm)	4.4 [2.8–4.8]	6 [5.5–8.4]	5.1 [4.8–5.2]
LAD (mm)	13.5 [11.4–15.4]	14 [12–19.4]	21.9 [16.0–27.3]
LA/Ao	1.4 [1.3–1.6]	1.3 [1.0–1.8]	2 [1.8–2.9]

Results are presented as median [range] for each variable for the cardiomyopathies (HCM, RCM) and control cats. Abbreviations: LAD, left atrial diameter; LA/Ao, left atrium to aorta ratio; LVWT, left ventricular wall thickness at end-diastole.

### Genetic variant discovery and statistical analyses

The 18 candidate genes studied are listed in Table 3.

**Table 3:** Feline cardiomyopathy target gene panel. Gene names and positions are based on FelCat9 assembly. *Genes exclusive to the 18-gene panel for cohort (i) across-breeds cat study.

Gene Acronym	Gene Name	Chromosome	Start Position	End Position
*MYL3*	*Myosin Light Chain 3*	A2	16,290,210	16,296,259
*TNNC1*	*Troponin C1*	A2	21,044,756	21,047,572
PRKAG2*	*Protein Kinase AMP-Activated * *Non-Catalytic Subunit Gamma 2*	A2	165,581,663	165,842,537
*CAV3*	*Caveolin 3*	A2	50,098,852	50,115,062
PDLIM3*	*PDZ and LIM Domain 3*	B1	16,445,054	16,474,763
*PLN*	*Phospholamban*	B2	109,748,495	109,748,653
*MYH7*	*Myosin Heavy Chain Gene 7*	B3	76,134,518	76,188,380
*TPM1*	*Tropomyosin 1*	B3	43,987,226	44,036,037
*ACTC1*	*Actin Alpha Cardiac Muscle 1*	B3	70,080,059	70,085,659
*MYH6*	*Myosin Heavy Chain 6*	B3	76,134,518	76,188,380
*MYBPC3*	*Myosin Binding Protein C*	D1	101,324,989	101,341,953
*CSRP3*	*Cysteine And Glycine Rich Protein 3*	D1	76,776,148	76,804,617
*ACTN2*	*Actinin Alpha 2*	D2	12,369,479	12,442,749
*MYL2*	*Myosin Light Chain 2*	D3	8,973,170	8,980,956
*TCAP*	*Titin-Cap (Telethonin)*	E1	40,752,572	40,753,322
*TNNI3*	*Troponin I3*, *Cardiac Type*	E2	3,439,821	3,450,055
*TNNT2*	*Troponin T2*, *Cardiac Type*	F1	42,194,772	42,209,527
*GLA*	*Galactosidase Alpha*	X	83,631,654	83,639,229
*LAMP2*	*Lysosomal Associated Membrane Protein 2*	X	100,973,530	101,012,558

### Study 1: Across-breeds

**Fig. 1:**
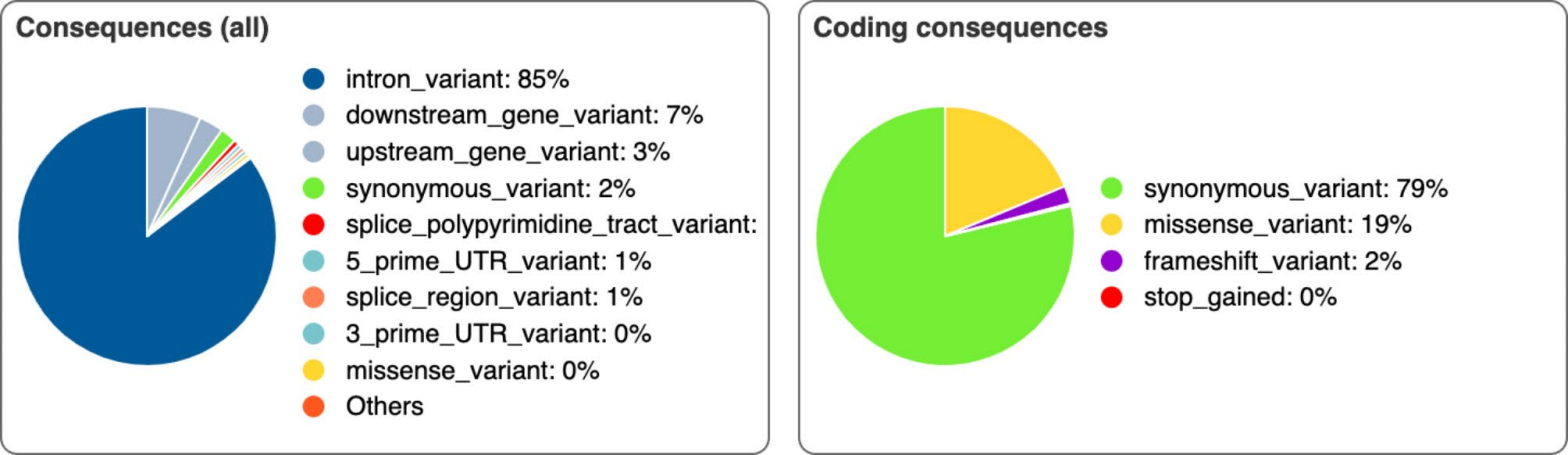
Statistical analysis of variants identified in the Across-breeds cat cohort using targeted next-generation sequencing of 18 cardiomyopathy-related genes, annotated with the Ensembl Variant Effect Predictor tool. The gene selection was based on human cardiomyopathy literature.

Targeted next-generation sequencing analysis revealed the presence of a total of 4025 variants, single nucleotide variants (SNVs) (3400) and indels (666) in the 18 candidate genes in the Across-breeds cohort. Details of the identified genetic variants are presented in Fig. 1.

#### Chi-squared comparisons

The annotation of the identified variants detected eight high-impact variants, including the known Ragdoll missense variant R820W in *MYBPC3*^15^, which was included as a positive control. However, none of these variants had a significantly different prevalence between cases and controls when analysed both within and across the cat breeds.

Comparisons using the Chi-squared (χ²) test of the allelic and genotypic frequencies of the identified variants with a predicted moderate or modifier effect on the encoded protein identified a total of 160 variants with a significant (*P* ≤ 0.05, nominal P-value) difference between HCM cases and controls. 157 of these variants were intronic: 91/157 had a higher prevalence in cases, with 8/157 being present only in cases (see Supplementary Table 1 for full list of variants with a statistically significant difference). Four of these variants remained significant after correcting for multiple testing: one intronic variant (X:101006474 T > C, *P* = 0.0002) in lysosomal associated membrane protein (*LAMP2)* gene, one 3’UTR (F1:42194903 G > GGT, *P* = 0.025) located in *TNNT2* gene and two missense variants (B3:76167563 C > T L88F, *P* = 0.003; D1:76804158 T > C I45V, *P* = 0.004) located within the novel gene *ENSFCAG00000040035* (overlapping *MYH7*) and cysteine and glycine rich protein *(CSRP3)* gene respectively. The intronic SNV in *LAMP2* was only present in control cats. The 3’UTR variant located in *TNNT2* had a significantly higher genotypic frequency in cases. According to the Softberry FPROM human promoter predictor analysis close to this 3’UTR SNV there are two promoters. However, this SNV did not directly overlap with these promoter predictions. Moreover, this 3’UTR SNV exhibited a high effect allelic frequency (EAF) of 0.60 in the general cat population, as evidenced by data from the 99Lives Cat Whole Genome Sequencing (WGS) Initiative database (https://cvm.missouri.edu/research/feline-genetics-and-comparative-medicine-laboratory/99-lives/)^30^.

The two missense variants were present in both case and control cats with a higher prevalence in controls and were located within the novel gene *ENSFCAG00000040035* (overlapping *MYH7*) (B3:76167563 L88F) and *CSRP3* gene (D1:7680415 I45V) respectively. The *CSRP3* gene missense variant has a reported ‘sorting intolerant from tolerant’ (SIFT) score of 0.34, which is considered ‘tolerated’. Analysis of the 99Lives cat WGS database revealed that both variants were prevalent in the general cat population.

#### Genomic association analysis

**Fig. 2:**
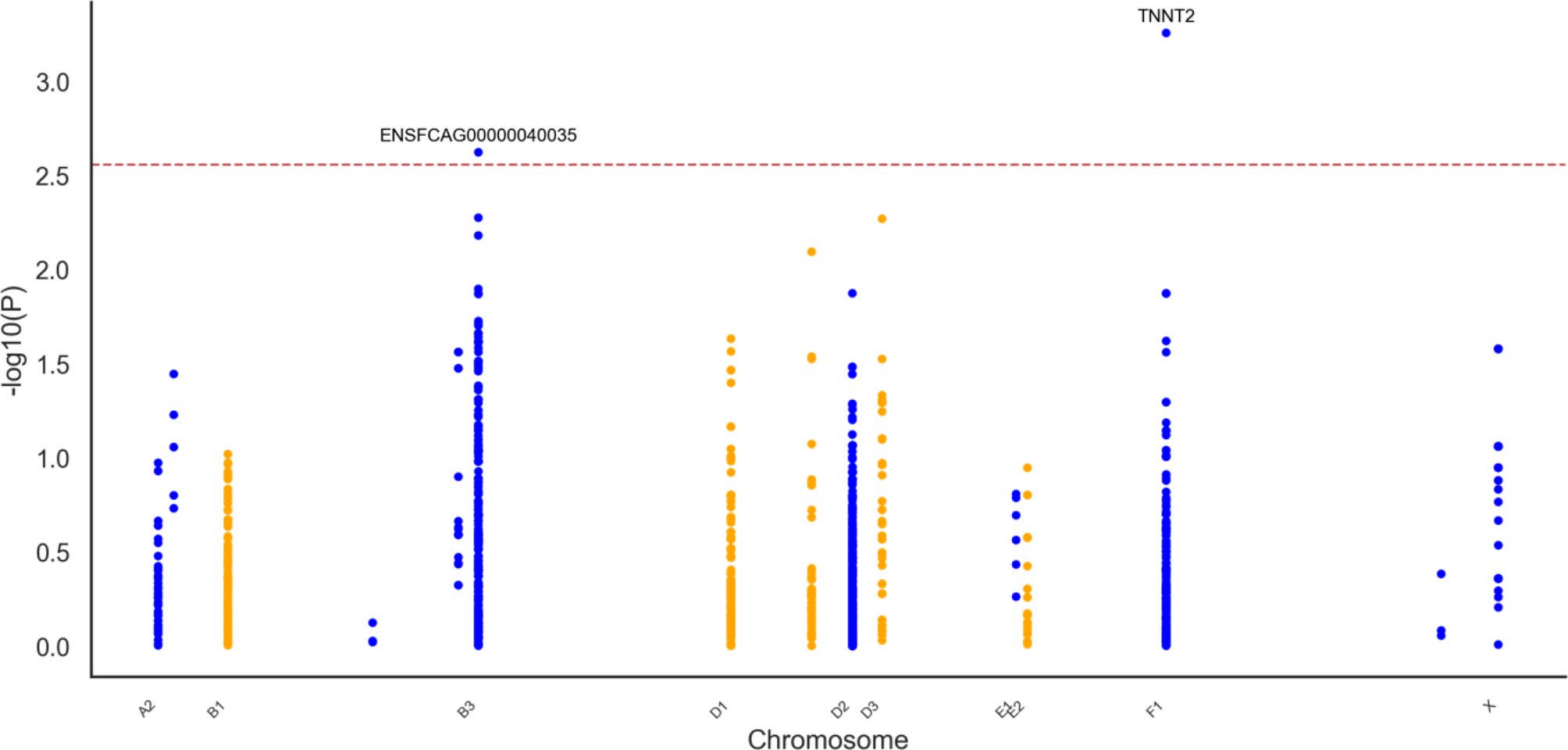
Manhattan plot presenting the results of the genomic association analysis for hypertrophic cardiomyopathy susceptibility in the Across-breeds cat cohort. Genomic location (x-axis) is plotted against -log10(P-value) (y-axis). The red line indicates the significance threshold (*P* ≤ 0.05) after multiple testing correction.

The genomic association analysis for the Across-breeds cohort which included age, sex and breed as covariates, identified one intronic variant in *TNNT2* (F1:42199381 CA > C) significantly associated with HCM (*P* = 0.0006). This variant was exclusively present in affected individuals (*n* = 5). The Softberry NSITE analysis predicted that this variant affected the motifs that were able to bind to the nearby transcription factor binding sites (TFBS), with three additional motifs identified in the intronic sequence compared to the control cat population sequence (see supplementary Table 8). The Manhattan plot presenting the results of the genomic association analysis for HCM susceptibility in the Across-breeds cat cohort is shown in Fig. 2. Moreover, this intronic variant had a low frequency (EAF = 0.026) in the general cat population, as represented in the 99Lives cat WGS database analysis.

### Study 2: Birman cats

**Fig. 3:**
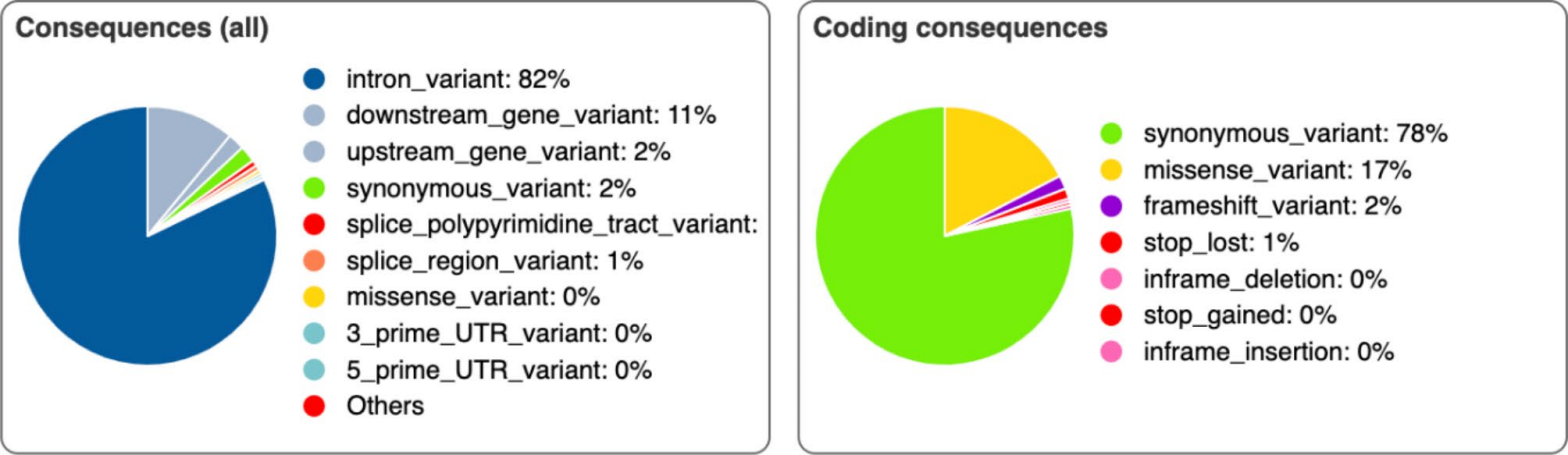
Statistical analysis of variants identified in the Birman pedigree cat cohort using targeted next-generation sequencing of 16 cardiomyopathy-related genes, annotated with the Ensembl Variant Effect Predictor tool. The gene selection was based on human cardiomyopathy literature.

The targeted next-generation sequencing analysis of the Birman cat cohort revealed the presence of 2298 genetic variants, SNVs (1921) and indels (395) across the candidate genes. Details of the identified genetic variants are presented in Fig. 3.

#### Chi-squared comparisons

The annotation of the genetic variants revealed five high-impact variants within the Birman cohort. However, their frequency was not significantly different between case and control cats. When variants with a predicted moderate or modifier effect were compared using the χ² test, 177 variants were identified with significantly different frequencies (*P* ≤ 0.05, nominal P-value) between cases (HCM and RCM) and controls. Among these variants was a missense SNV located in the *ENSFCAG00000040035* gene, resulting in a glutamic acid to glutamine substitution at position 22 (B3:76167365 G > C E22Q, *P* = 0.048). This variant was more prevalent in cases (heterozygous in five HCM and three RCM). Notably, this missense variant is synonymous with a variant in the *MYH7* gene and overlaps with the microRNA miR-208B (*ENSFCAG00000018043*), previously implicated in heart disease^31^. The variant exhibited a higher frequency in the case population (EAF = 0.29) compared to both the control Birman population (EAF = 0.14) and the general cat population (EAF = 0.20), as represented by the 99Lives cat WGS database. Moreover, one 3’UTR SNV (D1:76785090, C > A, *P* = 0.03) within *CSRP3* gene was identified only in control cats. This variant exhibited comparable frequencies (EAF = 0.14) in both the control Birman population and the 99Lives WGS general cat population. Nevertheless, the Softberry analysis did not reveal any further overlapping of this 3’UTR SNV with promoter regions. Fourteen intronic variants located in actin alpha cardiac muscle 1 *(ACTC1)*, *MYH7* and *TNNT2* genes were more prevalent in cases (*P* ≤ 0.05). Specifically, variants in the *ACTC1* gene exhibited an EAF of 0.18 in cases, while being absent in controls. Analysis of the 99Lives cat WGS database revealed these variants had an EAF of < 0.05 in the general cat population.

When cases were restricted to cats with HCM, the χ² comparisons revealed 153 variants that were significantly different (*P* ≤ 0.05) between cases and controls. Of these 153 variants, 117 had a higher prevalence in cases, with 78 intronic variants present only in cats with HCM (mainly spanning the genes *ACTC1* and actinin alpha 2 *(ACTN2)*).

Full details of the identified variants in the HCM and RCM analyses and the HCM analyses are presented in Supplementary Tables 3 and 4, respectively. The majority of variants in the *ACTN2* gene were identified exclusively in cases with an EAF of 0.13, while being absent in controls. Analysis of the 99Lives cat WGS database revealed these variants had a low EAF of < 0.05 in the general cat population.

#### Genomic association analyses

I. Genomic Analysis: HCM Cases (*n* = 8) and Controls (*n* = 14).

**Fig. 4:**
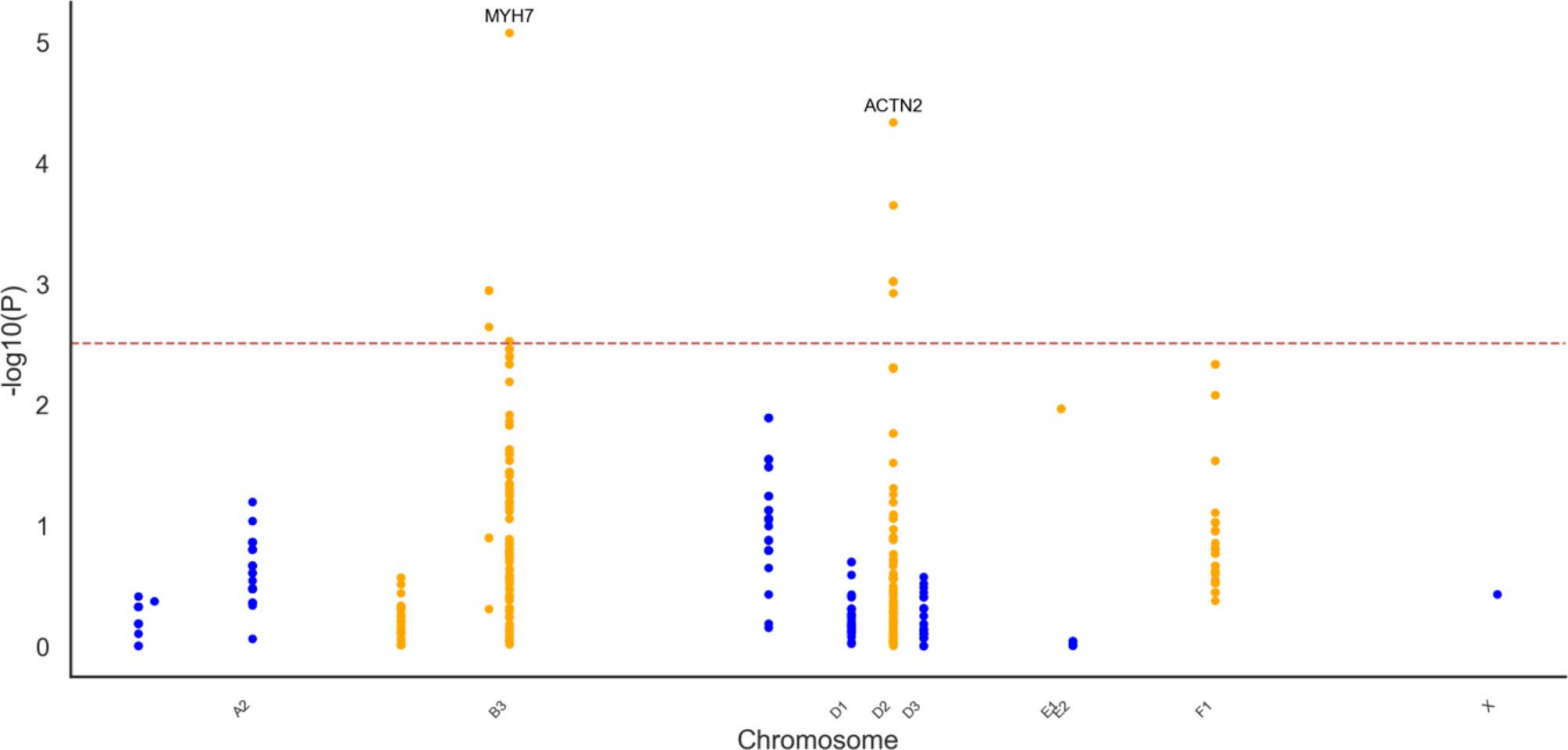
Manhattan plot presenting the results of the genomic association analysis for hypertrophic cardiomyopathy in Birman cats. Genomic location (x-axis) is plotted against -log10(P-value) (y-axis). The red line indicates the significance threshold (*P* ≤ 0.05) after multiple testing correction.

Genomic association analysis, restricted to HCM cases, and including age and sex as covariates, identified 24 intronic variants with a significant association with HCM. The top intronic variant for each gene associated to cases were located within *MYH7* (B3:76168426 G > A), *ACTN2* (D2:12374576 C > T*)* and *ACTC1* (B3:70080970 T > TC) genes. One intronic variant in *ACTN2* (D2:12407434 GGGGT > G) showed a significant association with controls. Analysis of the 99Lives cat WGS database revealed that variants more prevalent in Birman HCM cases had lower EAFs in the general cat population. For *MYH7* and *ACTN2*, the case EAFs were 0.38 and 0.31 respectively compared to a general population EAF of 0.13 for both variants. The *ACTC1* variant exhibited a case EAF of 0.18 and a general cat population EAF of < 0.05. The Manhattan plot presenting these results is shown in Fig. 4.

II. Genomic Analysis: HCM and RCM Cases (*n* = 14) and Controls (*n* = 14).

**Fig. 5:**
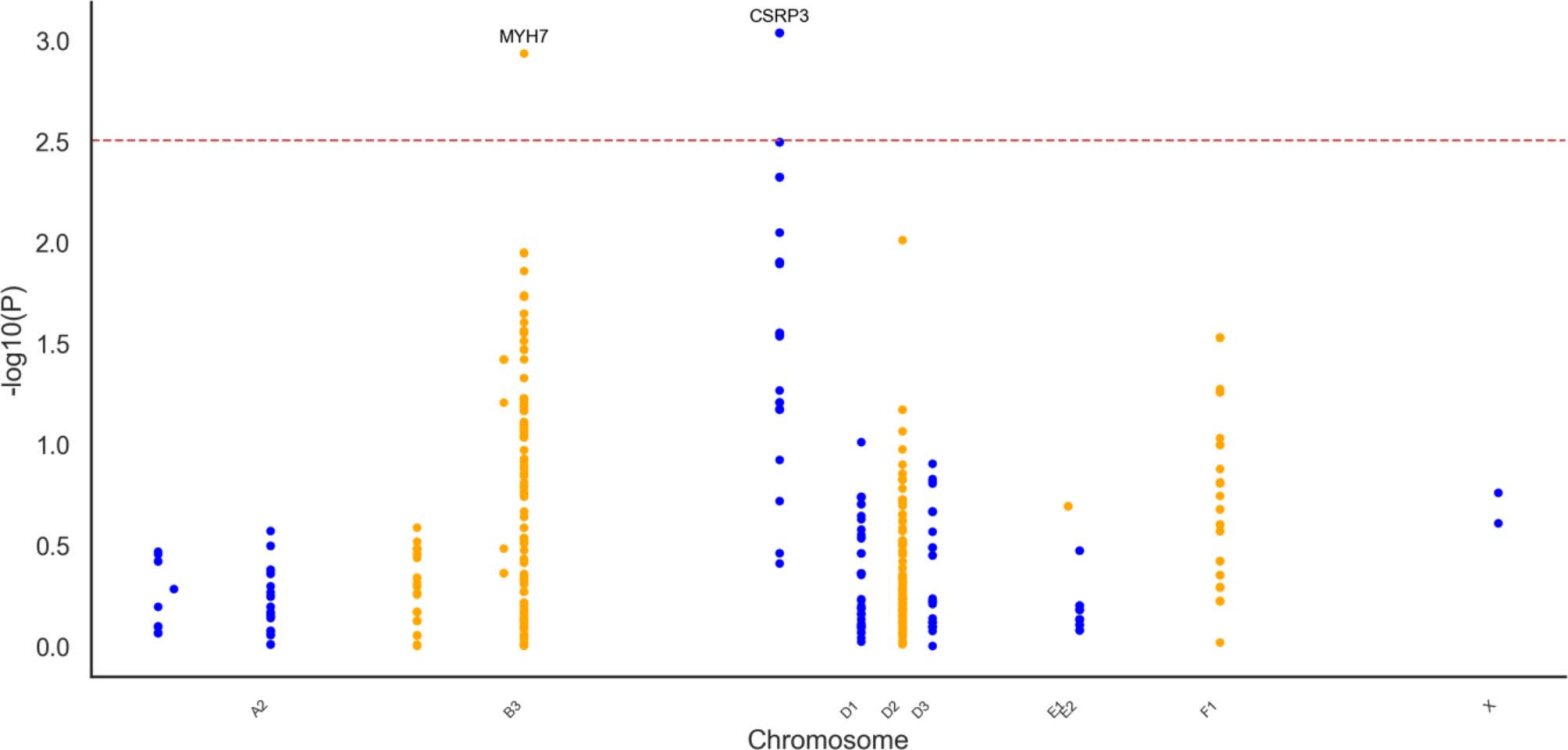
Manhattan plot presenting the results of the genomic association analysis for hypertrophic cardiomyopathy and restrictive cardiomyopathy in Birman cats. Genomic location (x-axis) is plotted against -log10(P-value) (y-axis). The red line indicates the significance threshold (*P* ≤ 0.05) after multiple testing correction.

The genomic association analysis of the merged HCM and RCM phenotypes identified ten intronic variants with a statistically significant difference (*P* ≤ 0.05) between cases and controls, nine within *CSRP3* and one within *MYH7* gene. Analysis of the 99Lives cat WGS database revealed nine of the variants more prevalent in the Birman control population had a high EAF (> 0.55) in the general cat population. The one SNV (*CSRP3* D1:76784404 G > A) more prevalent in Birman cases (EAF = 0.82) had a lower frequency (EAF = 0.26) in the general cat population. The Manhattan plot presenting these results is shown in Fig. 5.

## Discussion

We aimed to identify novel genetic variants associated with HCM and RCM in Birman cats, as well as across pedigree breeds and DSH cats. We performed two studies using a targeted cardiomyopathy gene panel on meticulously phenotyped cats. Although there are a few shared genetic variants associated with HCM resistance or susceptibility across cat breeds, the genetic architecture of the disease seems to be breed-specific.

Moreover, our studies identified high-impact variants within sarcomeric genes that were present at similar frequencies in both HCM cases and healthy controls. Similarly, several missense variants were present in both cases and controls, with no significant differences between the groups. These findings indicate that studies with appropriate sample sizes of cases and controls are required for the identification of truly causative HCM variants in cats. Nevertheless, we did identify a missense variant located on a novel gene *ENSFCAG00000040035*, which overlaps with *MYH7* and a microRNA (miR-208). This missense variant (E22Q) had a significantly higher prevalence in HCM cases (*P* = 0.048) in our Birman cat cohort. The function of *ENSFCAG00000040035* gene is unknown. In contrast, *MYH7* is a sarcomeric gene that encodes beta-myosin heavy chain; a key protein component of the sarcomere involved in cardiac contractile function^32^. Of increased interest is the presence of this variant within miR-208 (overlapping with *ENSFCAG00000040035* and *MYH7)*, which is expressed in cardiac tissue and regulates the production of beta-myosin heavy chain during cardiomyocyte development^33^. MicroRNAs (miRNA) are non-coding RNA sequences which are crucial in biochemical pathway regulation including during cardiac development^34^. According to previous studies, genetic variants in miRNA genes can have profound effects on miRNA functionality at all levels, including miRNA transcription, maturation, and target specificity, and as such they can also contribute to disease^35–37^. Therefore miR-208 might prove to be an important biomarker in feline cardiac disease, especially since it has already been linked to myocardial infarction, hypertensive heart disease^38,39^ and dilated cardiomyopathy in humans^31^. Relationships between miRNAs and cardiac conditions such as left ventricular hypertrophy and fibrosis have already been shown across studies^40,41^, including evidence for other miRNAs that overlap with the *MYH7* gene serving as biomarkers for human HCM^42,43^. Nevertheless, the role of microRNAs in feline HCM remains largely unknown. There is only one previous study that compared serum miRNA profiles between a healthy control cat cohort (*n* = 12) and cats with advanced HCM and documented clinical signs (ACVIM classification stage C) (*n* = 11)^44^. In that study, seven human HCM-associated miRNAs (causing cardiac tissue damage and disturbed blood flow) were upregulated in the HCM feline cohort^44^. These findings suggest that miRNAs might also play a role in feline HCM and could potentially be useful biomarkers as in humans. Notably, the miR-208 was not among the upregulated miRNAs within their feline cohort, suggesting our miR-208 variant may be specific to Birman pedigree cats. The missense variant (E22Q) in *ENSFCAG0000004003* exhibited a higher prevalence in the 99Lives general cat population compared to our Birman study control group. This finding suggests the variant may be specifically associated with HCM and RCM in Birmans, comparable to breed-specific variants previously identified in Maine Coons and Ragdolls. The lower frequency in the Birman controls relative to the general population could indicate a breed-specific effect. Alternatively, the higher frequency within the 99Lives database may reflect the inclusion of cats predisposed to cardiomyopathies, given the lack of comprehensive echocardiographic phenotyping for the majority of samples in this database. Further studies in Birman cats are needed to validate these results and confirm the potential role of miR-208 and/or *ENSFCAG00000040035* in feline HCM susceptibility.

Recent studies suggest intronic variants might play a more significant role in HCM susceptibility than previously thought^45,46^. Whole genome sequencing studies of human HCM have identified pathogenic intronic variants that disrupt splicing and TFBS^45^. In our study, we identified several intronic variants significantly associated with HCM susceptibility in cats, both across cat breeds and in the Birman cohort. Of particular interest was the intronic variant in *TNNT2* (position F1:42199381) which was present only in HCM cases in the Across-breeds cat study and had a very low frequency in the general cat population. Softberry analysis predicted that this intronic variant affects the number of motifs in or around the TFBS which has the potential to influence the binding of transcription factors, and consequently gene regulation. Several variants in *TNNT2* gene are associated with human HCM^47^. *TNNT2* encodes cardiac troponin-T (cTnT) a cardiac specific protein forming part of the troponin complex associated with thin filaments. This protein plays a crucial role in cardiomyocyte contraction and relaxation through its interaction with actin and tropomyosin^48^. Further studies are needed to confirm whether this intronic variant plays a regulatory role and contributes to HCM susceptibility since Softberry tools currently provide predictions based on the human (and not the feline) genome assembly. Moreover, we found several intronic variants associated with HCM susceptibility both in the Across-breeds and Birman cat studies, however it is difficult to assess if they have a pathological impact. Further genomic and functional genomic studies with larger sample sizes are needed to shed light on the role of intronic variants in feline HCM susceptibility.

We investigated RCM alongside HCM in Birman cats following previous unpublished observations of cats within the same family being diagnosed with different cardiomyopathy phenotypes, as has been observed in affected human families^49^. There are significant similarities in disease presentation between HCM and RCM, with both conditions leading to diastolic dysfunction and increased end-diastolic pressure^49^, predisposing cats to heart failure. RCM in cats shares similar histological features with HCM; thus, it has been proposed that RCM could be part of the HCM spectrum^50^. An intronic variant in the *CSRP3* gene was significantly associated with the combined group of HCM and RCM cases. The *CSRP3* gene encodes the muscle LIM protein (MLP) of functional importance for calcium handling and signalling in cardiomyocytes^51^. Variants within this gene have been identified as causative for both human HCM and dilated cardiomyopathy^52,53^. Within our Birman cat cohort, the intronic variant was more prevalent in the control population and may have a protective role. Our results indicate the potential presence of shared protective variants against the phenotypic expression of RCM/HCM phenotype within Birman cats. To identify whether our intronic variant is playing a protective role and understand the exact mechanism behind this, further molecular and functional studies would need to be conducted, alongside validation in another case-control study of Birman cats. Future studies on Birman cats should include related individuals exhibiting varying cardiomyopathies. Our Birman cohort included related cats, however these consisted of only four cats in the control population and two HCM cats.

Future research with larger sample sizes across multiple breeds, including Birmans, will further elucidate the role of specific genes and genetic variants in HCM susceptibility. Increasing the sample size will enable separate analyses to be conducted within each individual breed and among the DSH population, potentially revealing additional genetic variants of interest. Furthermore, incorporating genes of recent interest, such as junctophilin 2 (*JPH2*)^54^, within future analyses could provide valuable insights into the genetic basis of HCM.

## Conclusion

This study identified several genetic variants with a predicted high or moderate impact located in sarcomere candidate genes for feline HCM. However, these were not unique to affected cats, therefore were not considered causative of HCM. These findings support the need for genetic studies of adequate sample size to properly assess the role of genetic variants with a predicted high impact. Moreover, we identified several intronic variants with a significant association with HCM susceptibility or protection, highlighting that non-coding variants may also play a role in feline HCM. Furthermore, in Birman cats, we identified an exonic variant in a gene of unknown function *(ENSFCAG00000040035)* overlapping with *MYH7* and a myocardium related microRNA (miR-208). Further studies are needed to characterise the role of this variant in HCM susceptibility within this breed. Our findings suggest that feline HCM likely follows a complex polygenic or oligogenic inheritance pattern. The shared genetic variants between HCM and RCM in Birman cats indicate a breed-specific phenotypic overlap. These results highlight important genomic regions for further investigation. Future research should focus on functional characterisation of identified variants and exploration of microRNA roles in feline HCM.

## Materials and methods

### Ethical approval

Ethical approval was received by the Clinical Research Ethical Review Board of the Royal Veterinary College (URN 2019 1942-3, URN 2016 1515, URN 2015 − 1378), the study followed all relevant guidelines and regulations. The study is reported in adherence to ARRIVE (https://arriveguidelines.org) guidelines.

### Study population

Cats were recruited into the study following routine or invited echocardiographic screening for heart disease or following post-mortem examination conducted at the Royal Veterinary College (RVC). Of the cats included in the study total (*n* = 72), all were initially diagnosed with HCM through ante-mortem echocardiographic measurements. Subsequently 13 of these cats (two from the Birman cohort and 11 from the Across-breeds cohort) had their HCM diagnosis confirmed at necropsy. Cats without evidence of HCM or other cardiac disease (confirmed via echocardiography) and above the age of 9 years old were used as controls. Cats of any age with a confirmed HCM phenotype were used as cases. Among the Birman cohort, two control cats were littermates, two control cats shared the same dam, and two HCM cats shared the same dam.

Our work included two studies: (i) An Across-breeds cat study, totalling 44 phenotyped cats (controls = 23, HCM = 21) representing 21 non-pedigree cats (DSH) and pedigree breeds (4 Bengal, 8 British shorthair, 1 British longhair, 6 NFC, 3 Ragdoll, and 1 Maine coon). Among the HCM cases we included a Ragdoll cat confirmed homozygous for the HCM-associated *MYBPC3* variant (R820W); (ii) A Birman pedigree cat study, totalling 28 phenotyped Birman cats (controls = 14, cases = 14, including 8 cats with HCM, and 6 cats with RCM).

### Phenotyping

The cardiac phenotype was defined by echocardiography and/or gross pathology and histopathology, with owner consent. Echocardiography was performed by a board-certified veterinary cardiologist, or by a cardiology resident under the supervision of a board-certified veterinary cardiologist, using a Vivid E9 or Vivid I ultrasound machine (GE Systems, Hatfield, Hertfordshire, UK), with a 7.5 or 12 MHz phased-array transducer. Standard echocardiographic views were acquired, and video loops recorded^9^. All studies were measured off-line using dedicated echocardiographic software (EchoPac, GE Systems, Hatfield, Hertfordshire, UK).

On echocardiography, the thickness of the left ventricular free wall (LVFW) and interventricular septum (IVS) was measured by a leading edge to leading edge technique from a 2D right parasternal long-axis (RPLA) four- or five-chambered view, and a short-axis view at the papillary muscle level (RPSA). The thickest end-diastolic segment was averaged over three different cardiac cycles in each view (RPLA and RPSA). End-diastolic frames were defined as the first frame after mitral valve closure in RPLA and as the time point in the cardiac cycle of greatest left ventricular internal diameter in RPSA^9^. The greatest end-diastolic wall thickness of these measured views (RPLA septal, RPLA free wall, RPSA septal, RPSA free wall) was defined as LVWT and used for data analysis. Left atrial linear dimensions were measured as left atrial to aortic ratio (LA/Ao ratio) and left atrial diameter (LAD). The LA/Ao was measured as the ratio of the left atrium to aorta measured in 2D from an RPSA view at the heart base, in the frame after aortic valve closure^55^. The LAD was measured as the cranial-caudal LA dimension from a RPLA 4-chambered view, in the frame before mitral valve opening^56^. Left ventricular (LVFS%) fractional shortening was measured by M-mode from a right parasternal short-axis at the papillary muscle. Systolic anterior motion of the mitral valve (SAM) was assessed on colour Doppler and 2D echo from a right parasternal long-axis five-chamber view.

HCM was defined as LVWT ≥ 5.5 mm at end-diastole. Cases with concurrent disease that could contribute to LVH were excluded from the study. These conditions included systemic hypertension (systolic blood pressure > 160 mmHg)^57^, aortic stenosis or hyperthyroidism^58^. Healthy cats (control group) were defined as having a LVWT < 5.5 mm and aged ≥ 9 years old to minimise inclusion of cats with late onset HCM. Necropsy examinations were performed by a single trained observer (Lois Wilkie), and HCM was defined as a hypertrophied LV in the presence of myofiber disarray and interstitial/replacement fibrosis on histopathology^59^.

In the Birman cat cohort, in addition to HCM cases we also included RCM cases, defined as the presence of left or biatrial enlargement (left and/or right atrial diameter in RPLA view > 16 mm), LVWT ≤ 5.5 mm and normal left ventricular systolic function (LVFS% > 30%).

### Post-Mortem Diagnosis of Hypertrophic Cardiomyopathy (HCM) and Restrictive Cardiomyopathy (RCM) in Cats

#### Macroscopic Evaluation

Gross pathology reports were reviewed for body weight, cardiac weight, and ventricular wall thicknesses. Measurements of right ventricular free wall (RVFW), interventricular septum (IVS), and left ventricular free wall (LVFW) were taken on a transverse cross-section, one-third from apex to base. The expected RVFW: IVS: LVFW ratio was 1:3:3, with relative cardiac weight reference range of 0.28–0.88% of body weight^60^. HCM was characterised by increased cardiac weight and LVH.

#### Histopathological Examination

Cardiac tissues were formalin-fixed, paraffin-embedded, and stained with haematoxylin-eosin and Masson’s trichrome^60^. A single observer blindly evaluated slides using the Olympus BX51TF microscope at magnifications ranging from x10 to x100^60^. A semiquantitative scoring system was followed assessing: fibrosis (interstitial, perivascular, replacement, subendocardial); intramural arteriolosclerosis; myocyte degeneration; inflammatory cell infiltrate and fat infiltration (absent/present); and myocyte hypertrophy and disarray (graded 0–3)^60^.

### Blood and tissue collection and DNA extraction

Myocardial samples collected at necropsy were received from Birman breeders following death with suspicion of heart disease. For the Across-breeds cats, liver samples were obtained following routine necropsy examinations at the RVC. Residual blood (derived from clinical testing) was used for this project from blood collected by either a qualified veterinarian or veterinary nurse following echocardiography (using the same equipment and expert for each diagnosis) to exclude systemic diseases that could affect the heart and to measure cardiac biomarkers. DNA was extracted from whole blood/liver/myocardial samples using two commercial kits: DNeasy Blood and Tissue Kit (Qiagen^®^) and GeneJet Whole Blood Genomic DNA Purification Mini Kit (Thermo Scientific^®^) according to the manufacturers’ instructions. DNA quality and quantity were assessed using Denovix DS-11 Series spectrophotometer and Invitrogen Qubit 4 Fluorometer, respectively.

### Feline HCM gene panel and targeted next-generation sequencing (tNGS)

We developed a gene panel for feline HCM and RCM based on candidate genes previously implicated in human cardiomyopathies (Table 3). This feline panel was equivalent to the Illumina TruSight Cardio Panel^61^ which is applied in suspected cases of human cardiomyopathy. In the first study (Across-breeds cohort) we included a panel of 18 candidate genes (Table 3). The same panel was used in the second study (Birman cohort) with the exclusion of two genes. These two genes were excluded due to limited variation, with no exonic variation being identified in these genes from the first study.

### Targeted next-generation sequencing analysis

The raw sequencing data (FASTQ files) were assessed for quality control using FASTQC (v10.1)^62^ and trimmed to exclude adapter sequencing using Trimmomatic (v0.36)^63^ prior to mapping the reads on Felis Catus v9.0 genome assembly^64^ using the BWA aligner^65^. The matching variant file for the Felis Catus v9.0 genome assembly (Ensembl release version 95)^66^ was sorted against the reference dictionary to obtain known variant sites using Picard toolkit (v2.21.7)^67^. The reads were indexed, and duplicates removed using SAMTOOLS (v1.3)^68^. Base recalibration and variant calling to detect SNVs and indel variants were performed with the GATK (v.3.8) software^69^ using HaplotypeCaller^70^. Joint VCF files were created for cases and controls (for each study separately). Two separate VCF files for the Birman cases were created: one including both HCM and RCM cases and another only HCM. We ran a grouped analysis for cats with HCM and RCM phenotypes, as RCM has been suggested to be part of the HCM spectrum, i.e., these two phenotypes might represent diverse expressions of the same disease^49,50,71,72^. The SNV locations were obtained from Felis Catus v9.0 genome assembly using the Ensembl genome browser release version 95^73^. SNV annotation was performed using the Ensembl variant effect predictor (VEP) tool^74^.

The data from each study were analysed separately. Allelic and genotypic frequencies of genetic (SNV and indel) variants with a predicted high, moderate, or modifier functional impact according to VEP were compared between cases and controls to assess if there are statistically significant differences between the two groups. The Chi-squared test (χ2), with a significance level set at *P* ≤ 0.05 was used in this respect. A correction for multiple testing (0.05 divided by number of genes tested) was also applied.

To identify if any of the SNVs of interest in 3’UTR and other non-coding regions were located within a putative regulatory region we further interrogated these SNVs using Softberry software^75^. Specifically, to identify potential functional roles of our SNVs of interest we used BEDTools^76^ to extract SNV sequences 1500 bp either side of our SNV and ran comparisons against the corresponding 3000 bp sequence extracted from our sample containing the reference allele. These 3000 bp sequences were inputted into Softberry tools FPROM promoter predictor to look for predicted promoter regions in our significant 3’UTR SNVs and the NSITE tool to search for regulatory motifs in our 3’UTR and intronic regions^77^.

### Genomic association studies

A bed format genotypic file was generated from the VCF file for cases and controls using the PLINK software (v1.90)^78–80^. Each of the datasets was subjected to quality control (qc) measures using the following thresholds: call rate < 90%, minor allele frequency < 0.05, and Hardy-Weinberg equilibrium *P* < 10^− 6^. A genomic relationship matrix was created for all animals using the GEMMA (v0.98.1) algorithm^81^. GEMMA was used to run the genomic association analyses for HCM susceptibility using a mixed model where the genomic relationship matrix was included as a random effect to account for possible population stratification and age, sex, and breed as fixed effects in the first study (Across-breeds cat cohort), and lambda correction applied to the P-values. The same model with the exclusion of breed as a fixed effect was used in the second study (Birman cat cohort). The significance level was set at *P* ≤ 0.05 and a Bonferroni correction for multiple testing was applied. Python3^82^ in Jupyter notebook^83^ (for Mac OS) was used to create Manhattan plots to present the genomic analyses results.

### Allelic frequencies for variants of interest in the general cat population

The allelic frequencies of genetic variants associated with HCM and RCM were assessed in the general cat population using the 99Lives feline whole genome sequences (WGS) database, which includes data from 341 cats from random bred and pedigree breeds (https://cvm.missouri.edu/research/feline-genetics-and-comparative-medicine-laboratory/99-lives/)^30^. This comprehensive genomic resource constructed from diverse sampling of domestic cats provides robust estimates of variant prevalence in the broader feline population^30^. Notably, phenotyping for HCM or RCM is not part of the inclusion criteria for this database. Given the prevalence of these diseases in the cat population, it is plausible that several individuals within this dataset could be genetically predisposed to HCM or RCM.

## Electronic supplementary material

Below is the link to the electronic supplementary material.

Supplementary Material 1

## Data Availability

Sequence data that support the findings of this study have been deposited in the Sequence Read Archive (SRA) repository with the BioProject accession code PRJNA1083230. Upon publication, the data will be made fully accessible to the public without restrictions through the SRA repository. The data are currently available in read-only format via the following link: https://dataview.ncbi.nlm.nih.gov/object/PRJNA1083230?reviewer=c2989md3eb80b925ug7pmk265u.

## Abbreaviations

*ACTC1*: *Actin Alpha Cardiac Muscle 1*
*ACTN2*: *Actinin Alpha 2*
*ALMS1*: *Alstrom Syndrome 1*
ARVC: Arrhythmogenic Right Ventricular Cardiomyopathy
*CSRP3*: *Cysteine and Glycine Rich Protein 3*
DCM: Dilated Cardiomyopathy
DSH: Domestic Shorthair
HCM: Hypertrophic Cardiomyopathy
LA/Ao: Left Atrium to Aorta Ratio
LAD: Left Atrial Diameter
LVH: Left Ventricular Hypertrophy
LVWT: Left Ventricular Wall Thickness at end-diastole
*MYBPC3*: *Myosin-Binding Protein C*
*MYH7*: *Beta-Myosin Heavy Chain 7*
*MYL2*: *Regulatory Myosin Light Chain*
*MYL3*: *Essential Myosin Light Chain*
NFC: Norwegian Forest Cat
RCM: Restrictive Cardiomyopathy
SNV: Single Nucleotide Variant
tNGS: Targeted Next-Generation Sequencing
*TNNI3*: *Troponin I3, Cardiac Type*
*TNNT2*: *Troponin T, Cardiac Type*
*TPM1*: *Tropomyosin 1*
